# Effects of inflammatory responses, apoptosis, and STAT3/NF-κB- and Nrf2-mediated oxidative stress on benign prostatic hyperplasia induced by a high-fat diet

**DOI:** 10.18632/aging.102138

**Published:** 2019-08-14

**Authors:** Yongzhi Li, Benkang Shi, Fengming Dong, Xingwang Zhu, Bing Liu, Yili Liu

**Affiliations:** 1 Department of Urology, The Fourth Affiliated Hospital of China Medical University, Shenyang, Liaoning, China; 2 Qilu Hospital of Shandong University, Jinan, Shandong, China

**Keywords:** benign prostatic hyperplasia, high-fat diet, inflammatory responses, apoptosis, oxidative stress

## Abstract

This study determined whether or not benign prostatic hyperplasia (BPH) induced by a high-fat diet (HFD) is involved in inflammatory responses, apoptosis, and the signal transducer and activator of transcription (STAT3)/nuclear factor-kappa B (NF-κB)- and nuclear factor erythroid 2-related factor 2 (Nrf2)-mediated oxidative stress pathways. Forty rats were divided into four groups: control; HFD; testosterone; and HFD+testosterone. Hematoxylin and eosin (HE) staining was used to assess histologic changes. An enzyme-linked immunosorbent assay and Western blot analysis were used to detect levels of related proteins. Compared with the control group, the prostate levels of cyclooxygenase-2 (COX-2), inducible nitric oxide synthase (iNOS), tumor necrosis factor-α (TNF-α), interleukin-6 (IL-6), malondialdehyde (MDA), transforming growth factor-β1 (TGF-β1), and monocyte chemotactic protein-1 (MCP-1) were significantly increased, while the levels of glutathione peroxidase (GSH-Px), glutathione reductase (GR), glutathione (GSH), and superoxide dismutase (SOD) were decreased. The TNF-κB, Bcl-2, and caspase-3 levels were increased, while the Bax level was markedly decreased. The cytoplasmic expression of STAT3 and NF-κB was increased, while the nuclear expression of Nrf2 was markedly decreased compared with the control group. In summary, our results demonstrated that a long-term HFD might cause changes in inflammatory responses, apoptosis, and oxidative stress, thus contributing to prostatic hyperplasia. The underlying mechanisms might be related to the STAT3/NF-κB- and Nrf2-mediated oxidative stress pathway.

## INTRODUCTION

Benign prostatic hyperplasia (BPH), a chronic condition in aging men, is characterized by non-malignant enlargement of stromal and epithelial cells in the prostate [1, 2]. Although the etiology of BPH is not fully understood, some factors, such as hormonal disruption, inflammation, and oxidative stress, are clearly associated with the development of BPH [3, 4]. Recent studies have suggested that BPH is associated with metabolic syndrome, such as obesity, hyperglycemia, dyslipidemia, and hypertension, as well as urinary tract syndrome [5].

A high-fat diet (HFD) has been verified as one of the factors related to the activation of prostate cancer and BPH. Moreover, BPH has been identified as a new metabolic disease [6, 7]. Recent studies have demonstrated that obesity and hyperinsulinemia have positive effects on prostate volume and prostatic hyperplasia [8, 9]. A recent study indicated that HFD induces prostate fibrosis and inflammation [10]. Although many studies have affirmed the negative effects of HFDs on different systems, including the prostate, the molecular and morphologic mechanisms underlying proliferative disorders in the prostate are still unclear [11].

Recent studies have reported that HFD leads to oxidative stress and inflammation in the prostate gland [12, 13]. An imbalance between cell proliferation and apoptosis leads to continuous growth of epithelial and stromal cells, contributing to the development of BPH [14]. The connection between metabolic disorders and apoptosis/proliferation that occur in the course of BPH, requires further research. A diagnosis of diabetes and the level of obesity are regarded as independent risk factors for developing BPH [15].

This study determined the role of the inflammatory response, apoptosis, and oxidative stress in the process of BPH, and further verified the relationship of the signal transducer and activator of transcription (STAT3)/nuclear factor-kappa B (NF-κB)- and nuclear factor erythroid 2 related factor 2 (Nrf2)-mediated signaling pathways with BPH induced by HFD.

## RESULTS

### Prostate histologic alterations

**Figure 1:**
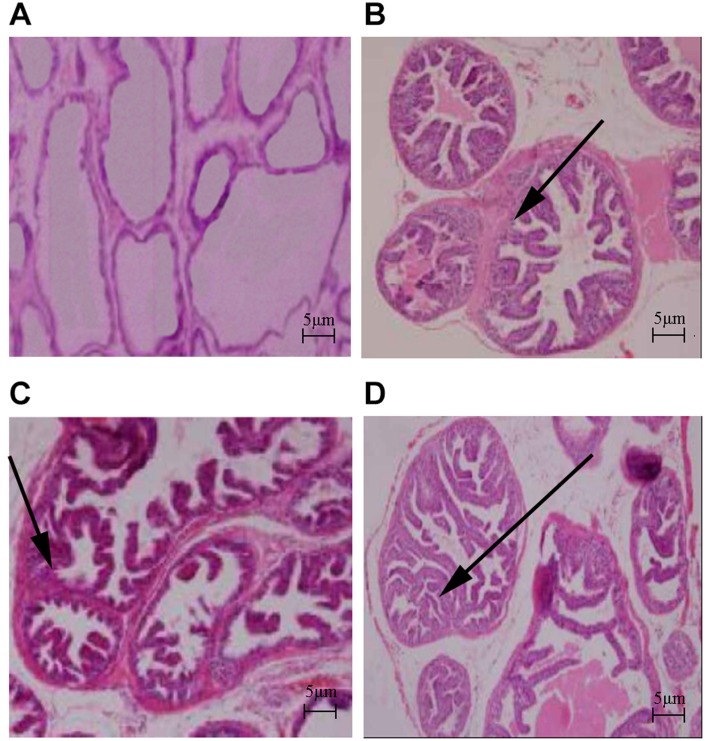
**Histologic changes in rat prostate (HE stain, ×40).** (**A**) Control group; (**B**) HFD group; (**C**) Testosterone group; (**D**) HFD+testosterone group.

The body weights of the HFD and HFD+testosterone groups were significantly higher than the control group (p<0.01). The prostatic wet weight of the control group was lower than the other three groups (p<0.01). The same results existed with respect to the prostatic index, which was significantly increased in the three groups compared to the control group (p<0.01; Table 1). As shown in Figure 1, the results of HE staining indicated that the control group presented with acini and typical characteristics, as well as high epithelial folds in the distal region of the gland. The HFD, testosterone, and HFD+testosterone groups exhibited various morphologic alterations in the ventral prostate; specifically, some areas showed apparent epithelial hyperplasia. The massive empty lipid vacuoles and some inflammatory foci were also demonstrated in the HFD and HFD+testosterone groups.

**Table 1:** Effects of a long-term HFD on body weight, prostatic wet weight, and the prostatic index.

**Groups**	**Body weight (g)**	**Prostatic wet weight (mg)**	**Prostatic index (mg/g)**
Control	458.92±32.5	256.32±15.6	0.56±0.11
HFD	495.56±29.5^##^	382.65±29.6^##^	0.77±0.16^##^
Testosterone	468.49±49.6	388.32±32.1^##^	0.83±0.21^##^
HFD+ testosterone	501.86±82.1^##^	420.21±27.9^##^	0.84±0.15^##^

Data are expressed as the mean ± SD. ^##^ indicates *P*<0.01 compared with the control group

### Levels of static inflammatory response-, apoptosis-, and oxidative stress-related proteins

**Figure 2:**
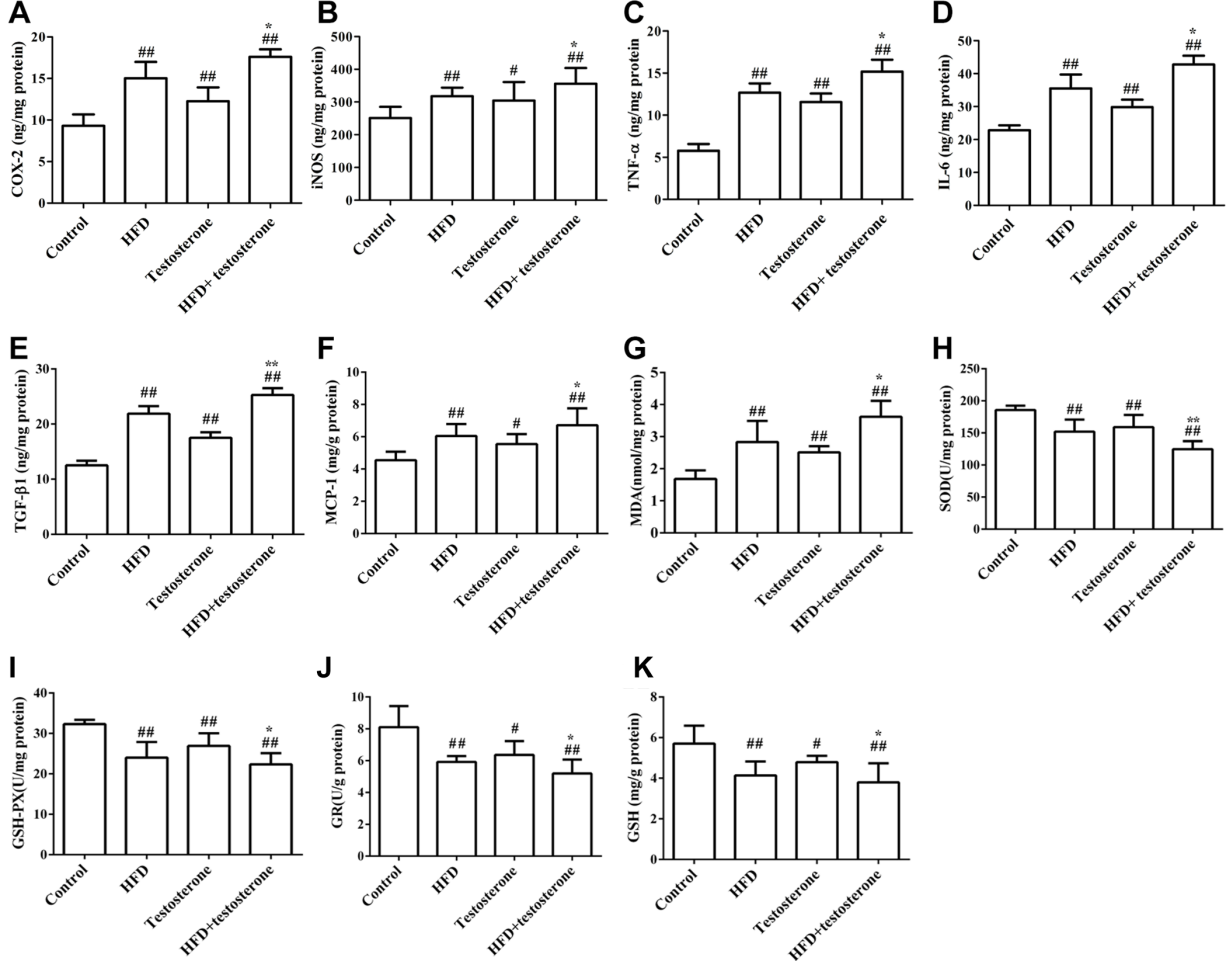
**Effects of a long-term HFD on prostatic COX-2, iNOS, TNF-α, IL-6, TGF-β1, MCP-1, MDA, SOD, GSH-Px, GR, and GSH** (**A**–**K**) levels by ELISA. Results are representative of three independent experiments. ^##^p<0.01 and ^#^p<0.05, compared with the control group; *p<0.05 compared with the testosterone group.

As shown in Figure 2, the levels of COX-2, iNOS, TNF-α, IL-6, TGF-β1, and MCP-1 were significantly increased compared with the control group, especially in the HFD+testosterone group (p<0.05 or p<0.01). These indicators were significantly altered in the HFD+testosterone group compared with the testosterone group (p<0.05 or p<0.01). The levels of SOD, GSH-Px, GR, and GSH were markedly reduced compared with the control group, while MDA was significantly increased (p<0.05 or p<0.01), especially in the HFD+testosterone group. In addition, these indicators were significantly altered in the HFD+testosterone group compared with the testosterone group (p<0.05 or p<0.01). All of the results indicated that HFD was related to the levels of static inflammatory response-, apoptosis-, and oxidative stress-related proteins.

### Effects of HFD on the expression of NF-κB, Bcl-2, caspase-3, Bax, STAT3, NF-κB p65, and Nrf2 protein, as detected by ELISA

**Figure 3:**
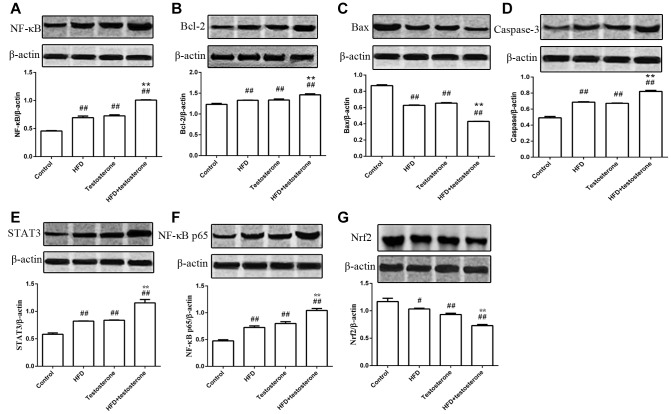
**Effects of a long-term HFD on the expression of NF-κB, Bcl-2, Bax, caspase-3, STAT3, NF-κB, p65, and Nrf2 protein.** ^##^p<0.01 and ^#^p<0.05 compared with the control group; *p<0.05 compared with the testosterone group.

As shown in Figure 3, the expression of NF-κB, Bcl-2, and caspase-3 protein were significantly increased compared with the control group, especially in the HFD+testosterone group (p<0.01), while Bax was markedly decreased (p<0.01). The expression of STAT3 and NF-κB p65 protein was dramatically increased compared with the control group (p<0.01), while Nrf2 was significantly decreased (p<0.01 or p<0.05), especially in the HFD+testosterone group. In addition, these indicators were significantly altered in the HFD+testosterone group compared with the testosterone group (*p*<0.01).

## DISCUSSION

BPH is a prevalent and chronic progressive disease that may be correctly defined as prostate gland enlargement secondary to hyperproliferation of stromal and glandular cells [16]. Several parameters, including inflammatory mediators, inflammatory genes, and oxidative stress, are considered to play a role in the development of BPH [17]. In the case of inflammation, the production of ROS is increased and can exhaust the anti-oxidative protection system [18]. ROS may also indirectly induce the formation of DNA adducts by initiating autocatalytic lipid peroxidation, generating a large variety of potential genotoxic breakdown products, such as MDA [19]. Measurement of MDA levels in plasma or serum provide a convenient *in vivo* index of lipid peroxidation, and represent a non-invasive biomarker of oxidative stress [20].

Disordered levels of growth factors, such as inflammatory cytokines, have been reported to be closely associated with the development of metabolic diseases [21]. NF-κB, a redox-sensitive transcriptional factor, is stimulated by oxidative stress, which might control the release of inflammatory cytokines [22]. In addition, testosterone is believed to accelerate the production of other inflammatory cytokines, such as COX-2, iNOS, TNF-α, and IL-6 [23]. The prostatic chronic inflammation state plays a vital role in the progression of BPH and prostatic carcinoma [24]. The levels of COX-2, iNOS, TNF-α, and IL-6 were clearly increased in rats given HFD. All of these findings suggested that growth factors and inflammatory cytokines played important roles in BPH induced by HFD.

It is well-known that inflammation has an effect on apoptosis. Chronic inflammation leads to proliferation in prostate tissues by modifying the expression of apoptotic protein. Apoptosis is of great importance in the regulation of cellular growth and tissue homeostasis, and is involved in a number of changes at the cellular level that lead to the death of functionally-impaired cells [25]. Analysis of the mechanism underlying apoptosis could be a vital part of BPH treatment. Previous studies have indicated that increased prostate volume is not due to excessive proliferation of prostate tissue, but is instead related to a decrease in prostate tissue apoptosis [26, 27]. COX-2 either exerts a pro-inflammatory or proliferative effect on prostate cells [28]. In the current study, the expression of Bcl-2 was significantly increased in the prostate tissues of rats treated with HFD or testosterone. The Bcl-2 protein family is divided into several groups based on structure and function, including proteins (such as Bcl-2) and pro-apoptotic proteins (such as Bax) [29]. Bcl-2 was the earliest discovered apoptosis suppressor gene. The mechanism by which Bcl-2 blocks apoptosis might be associated with antagonism of Bcl-2 and the apoptosis-promoting gene, Bax, thus inhibiting the release of cytochrome C from mitochondria to the cytoplasm and activating caspase protease [30, 31]. Bcl-2 expression were significantly increased after treatment with HFD+testosterone, while Bax expression was significantly reduced. In addition, the expression of caspase-3 was clearly increased. These findings showed that prostate hyperplasia induced by HFD might exert anti-apoptotic effects by increasing the expression of Bcl-2 and caspase-3 and decreasing the expression of Bax.

Our study showed that the MDA level was significantly increased in the prostate tissues of the HFD group, suggesting enhanced oxidative stress in the HFD treatment group. ROS production can suppress the anti-oxidant ability of the liver, including anti-oxidative enzymes and anti-oxidants [32]. Generally, BPH is accompanied by a down-regulation of anti-oxidants, such as GSH, GSH-Px, GR, and SOD [33]. GSH is a very important anti-oxidant and has a strong scavenging effect on free radicals. SOD and GSH-Px are the anti-oxidant enzymes involved in endogenous defense mechanisms against increased ROS. Of these, SOD serves as an important symbol to show anti-oxidant activity in patients with BPH [34]. Our results showed that the level of SOD was markedly reduced. Moreover, the results of GSH, GSH-Px, and GR were markedly reduced in rats fed HFD, which suggested that anti-oxidant systems were reduced after treatment with HFD. All of these findings indicated that HFD might reduce the anti-oxidant system, resulting in enhanced oxidative stress.

STAT3 is a key factor that activates NF-κB, and this activation contributes to the transcriptional regulation of inflammatory cytokines, such as TGF-β1, IL-6, iNOS, and MCP-1 [35]. Our results showed that the expression of STAT3 and NF-κB was significantly increased, and the levels of TGF-β1, IL-6, iNOS, and MCP-1 were also markedly increased in rats fed HFD. In a previous study, Stat-3 was involved in the activation of NF-κB in the prostate as a result of HFD feeding, leading to inflammation [36]. NF-κB is a redox-sensitive transcriptional factor stimulated by oxidative stress and plays an important role in producing the inflammatory cytokines [37, 38]. These results suggested that HFD increases the expressions of STAT3 and NF-κB, thus leading to the release of inflammatory factors. Nrf2 is a redox-sensitive transcription factor that regulates the expression of anti-oxidative genes [39, 40]. HFD increases the risk of developing BPH and prostate cancer through an influence on the NF-kB and Stat-3 signaling pathways [41]. The expression of Nrf2 was also significantly decreased in HFD, suggesting that HFD might reduce anti-oxidant activity by inhibiting the expression of Nrf2.

In conclusion, inflammatory cytokines (iNOS, COX-2, TNF-α, and IL-6), apoptosis-related proteins (Bcl-2 and Bax), and oxidative stress-related proteins (GSH-Px, GR, GSH, and SOD) were changed significantly in the HFD, testosterone, and HFD+testosterone groups compared with the control group. STAT3 and NF-κB were significantly increased and Nrf2 was significantly decreased, indicating that HFD might reduce anti-oxidant activity by inhibiting the expression of Nrf2 and be involved in STAT3/NF-κB- and Nrf2-mediated oxidative stress pathways. However, this study was only conducted in a rat model, thus a clinical study should be carried out to investigate the effects of HFD and testosterone on BPH.

## MATERIALS AND METHODS

### Materials

A total of 40 healthy male SD rats (8 weeks old; 250±10 g) were obtained from Shanghai SLAC Laboratory Animal Co., Ltd. (Shanghai, China) and maintained in a temperature- (22–24°C) and humidity-controlled room (55–60%). The rats were allowed free access to food and water and acclimated to the laboratory environment for 3 days prior to the study. All experimental procedures were conducted in conformity with the Institutional Guidelines for the Care and Use of Laboratory Animals, and protocols were approved by the Institutional Animal Care and Use Guidelines of China Medical University (Shenyang, China). These rats were divided into four groups: control group (n=10); HFD group (n=10), treated with HFD (carbohydrate, 29%; protein, 16%; fat, 55%) for 12 weeks; testosterone group (n=10), treated with testosterone (10 mg/kg/d) for the last 4 weeks; and HFD+testosterone group (n=10), treated with HFD for 12 weeks plus testosterone (10 mg/kg/d) for the last 4 weeks. All of the rats were sacrificed and the prostate glands were collected for parameter measurements.

### Histologic examination

The prostate glands were fixed in 10% formaldehyde solution and embedded in paraffin for hematoxylin and eosin (HE) staining, then examined under a light microscope. The body weight, prostatic wet weight, and prostatic index (rostatic wet weight (mg)/body weight (g)) of each group were measured.

### Measurements of inflammatory response-, apoptosis-, and oxidative stress-related proteins

To measure the prostatic cyclooxygenase-2 (COX-2), inducible nitric oxide synthase (iNOS), tumor necrosis factor-α (TNF-α), interleukin-6 (IL-6), malondialdehyde (MDA), glutathione peroxidase (GSH-Px), glutathione reductase (GR), glutathione (GSH), monocyte chemotactic protein 1(MCP-1), and superoxide dismutase (SOD) levels, partial prostatic tissues were immediately put into ice-cold normal saline containing 50 U/ml aprotinin. The tissue homogenate (10%, w/v) was prepared and centrifuged at 1200 × g for 10 min. The supernatant then obtained was used for COX-2, iNOS, TNF-α, TGF-β1, IL-6, iNOS, and MCP-1 measurements according to the ELISA method following the manufacturer's instructions on a VersaMax plate reader (Molecular Devices, city, CA, USA).

### Western blot analysis for protein expression

The western blot assay was used to measure the expression of Nrf2, Bcl-2, Bax, Caspase-3, STAT3, NF-κB, NF-κB-p65, and β-actin protein. In brief, a 70-mg aliquot of protein from each sample was loaded on 10% SDS-polyacrylamide gel, separated by electrophoresis under constant current, and subsequently transferred to nitrocellulose membranes (Millipore, Billerica, MA, USA). The membranes were blocked with 5% skim milk at room temperature for 1.5, then incubated with the primary antibodies for NF-κB-p65 (1:500 dilution), Bcl-2 (1:2000 dilution), Bax (1:1000 dilution), caspase-3 (1:1000), STAT3 (1:1000 dilution), Nrf2 (1:500 dilution), and β-actin (1:1000 dilution) at 4 C overnight. Next, the membranes were washed and incubated with the fluorescent secondary antibody at room temperature for 1 h. The protein blots were analyzed by densitometry using an Odyssey infrared imaging system and Image J software, and the relative ratio of the protein of interest was subjected to β-actin.

### Statistical analysis

Data were expressed as the mean ±standard deviation. The significance of differences among groups for the quantitative index was determined using one-way ANOVA, followed by a *post hoc* LSD test. The hepatic histopathologic evaluation was performed using the Mann-Whitney U test. The statistical analysis was conducted using SPSS 19.0 software, and a *p*<0.05 was considered statistically significant.
